# A structured expressive writing activity targeting body image-related distress among head and neck cancer survivors: who do we reach and what are the effects?

**DOI:** 10.1007/s00520-021-06114-y

**Published:** 2021-03-18

**Authors:** Heleen C. Melissant, Femke Jansen, Simone E. J. Eerenstein, Pim Cuijpers, Birgit I. Lissenberg-Witte, Kerry A. Sherman, Ellen T. M. Laan, C. René Leemans, Irma M. Verdonck-de Leeuw

**Affiliations:** 1 Department of Otolaryngology-Head and Neck Surgery, Amsterdam UMC, Vrije Universiteit Amsterdam, de Boelelaan 1117, 1081 HV Amsterdam, the Netherlands; 2 Cancer Center Amsterdam (CCA), Amsterdam UMC, Vrije Universiteit Amsterdam, Amsterdam, The Netherlands; 3 Department of Clinical, Neuro- and Developmental Psychology, Faculty of Behavioral and Movement Sciences, Amsterdam Public Health Research Institute, Vrije Universiteit Amsterdam, Amsterdam, The Netherlands; 4 Department of Epidemiology and Biostatistics, Amsterdam UMC, Vrije Universiteit Amsterdam, Amsterdam, The Netherlands; 5 Centre for Emotional Health, Department of Psychology, Macquarie University, Sydney, Australia; 6 Department of Sexology and Psychosomatic OBGYN, Amsterdam UMC, Academic Medical Center, Amsterdam, The Netherlands

**Keywords:** Body image, Head and neck cancer, Health-related quality of life, Self-compassion, Expressive writing

## Abstract

**Purpose:**

The aim of this pretest–posttest study was to investigate the reach and effects of My Changed Body (MyCB), an expressive writing activity based on self-compassion, among head and neck cancer (HNC) survivors.

**Methods:**

This pilot study had a pretest–posttest design. HNC survivors received an invitation to complete a baseline survey on body image-related distress. At the end of the survey, HNC survivors were asked if they were interested in the intervention study. This entailed the writing activity and a survey 1 week and 1 month post-intervention. The reach was calculated by dividing the number of participants in the intervention study, by the number of (1) eligible HNC survivors and (2) those who filled in the baseline survey. Linear mixed models were used to analyze the effect on body image-related distress. Logistic regression analysis was used to investigate factors associated with the reach and reduced body image-related distress. MyCB was evaluated using study-specific questions.

**Results:**

The reach of MyCB was 15–33% (depending on reference group) and was associated with lower education level, more social eating problems, and fewer wound healing problems. Among the 87 participants, 9 (10%) showed a clinically relevant improvement in body image-related distress. No significant effect on body image-related distress was found. Self-compassion improved significantly during follow-up until 1 month post-intervention (p=0.003). Users rated satisfaction with MyCB as 7.2/10.

**Conclusion:**

MyCB does not significantly improve body image-related distress, but is likely to increase self-compassion, which sustains for at least 1 month.

**Supplementary Information:**

The online version contains supplementary material available at 10.1007/s00520-021-06114-y.

## Introduction

Head and neck cancer (HNC) survivors have a high risk of body image-related distress (distress related to bodily changes [1]), since they often have to deal with body changes that cannot be easily hidden. Surgical treatment may lead to scars, disfigurements, an affected facial contour and expression (despite reconstructive surgery), and for some, living with a tracheostomy [2, 3]. Radiotherapy may result in fibrosis [4]. Surgery and radiotherapy may also induce lymphedema in the head and neck region, which is associated with body image-related distress [5]. Moreover, functional problems may occur that can negatively influence body image, such as speech problems or difficulties with eating [6]. A changed face can have profound personal and social consequences, affecting one’s identity and social life [2, 7]. Sexual concerns may also be present, for example, because patients have a diminished feeling of sexual attractiveness [3] It is estimated that 13–20% of HNC patients develop body image-related distress because of their changed body [8]. Body image is defined as “thoughts, feelings and perceptions about the entire body and its functioning” [9]. HNC patients with body image-related distress have a decreased health-related quality of life (HRQOL) and increased symptoms of depression [10, 11].

Despite the profound risk of body image-related distress in HNC patients, no effective interventions are available for this particular population. A systematic review on body image in HNC patients [12], found two studies assessing interventions to manage body image-related distress. The interventions focused on cosmetic restoration. No effects were demonstrated, compared to a control group.

To reduce body image-related distress, an intervention called “My Changed Body” (MyCB) was developed and tested among breast cancer survivors [13]. MyCB is an online writing activity that makes use of two elements: self-compassion and expressive writing. Self-compassion involves being kind to oneself and express self-kindness when suffering [14]. Stimulating self-compassion might improve people’s body image [15], especially in painful situations that are related to failure, humiliation or feelings of loss or rejection [14, 16], and provides a buffer against negative thoughts and feelings about the body [17]. Research among cancer survivors has shown that self-compassion is inversely related to both body image-related distress and psychological distress [18], and it may mediate the association between body image-related distress and psychological distress [19]. The other element in MyCB is guided expressive writing with a self-compassion focus. This entails asking individuals to choose a traumatic or upsetting experience and to write about their deepest thoughts and feelings [20], guided by specific prompts focused on self-compassion. Expressive writing may improve physical and psychological health outcomes [21]. A randomized controlled trial (RCT) among 306 breast cancer survivors demonstrated that MyCB was significantly more effective in reducing body image-related distress and psychological distress, and in improving self-compassion, compared to unstructured expressive writing [1].

The main objective of this study is to investigate the reach and effects of MyCB among HNC survivors. It is hypothesized that we will reach 13–24% of HNC survivors [10, 22, 23], and that MyCB will reduce body image-related distress, compared to pre-intervention levels. Possible factors associated with the reach are explored: sociodemographic and clinical characteristics, body image-related distress, body appreciation, self-compassion, psychological distress, HRQOL, HNC symptoms, and sexuality. Furthermore, possible associations between reduced body image-related distress post-intervention and sociodemographic and clinical characteristics are investigated.

## Methods

### Participants and procedures

Between September 2018 and September 2019, eligible HNC survivors (no thyroid cancer survivors) from the Department of Otolaryngology—Head and Neck Surgery at Amsterdam UMC, location VUmc, were recruited to participate in this study. The local ethics committee of VU University Medical Center decided that, according to the Dutch Medical Research Involving Human Subjects Act, ethical approval was not necessary as survivors were not subjected to procedures or required to follow rules of behavior. All participants signed informed consent.

HNC survivors were eligible if they: (1) received treatment for HNC with curative intent; (2) completed treatment 6 weeks to 5 years prior; (3) provided written informed consent. Exclusion criteria were: <18 years old, cognitive impairments (as mentioned in the patient’s medical file), inability to read and write Dutch, and participation in a prospective cohort study among HNC patients. The inclusion criteria did not include a screening test on body image, so that any HNC survivor with a need for body image care could participate in this study.

This non-randomized study consists of two parts. The first part is a cross-sectional survey on body image-related distress. HNC survivors who fulfilled the in- and exclusion criteria received an invitation letter from their physician to complete this paper-based survey (T0). The second part is a pretest–posttest study. At the end of the T0 survey, participants were asked if they were interested in an intervention study to evaluate MyCB to reduce body image-related distress. Interested participants received more information on the study and MyCB, and signed a second informed consent form. They could indicate their preference for using a booklet or website. After receiving the signed form, the researcher provided HNC survivors access to MyCB by sending the booklet or providing login instructions for the website. Participants also completed a paper-based survey 1 week (T1) and 1 month (T2) post-intervention.

### Intervention “My Changed Body”

MyCB was developed and researched in Australia targeting breast cancer survivors [13]. In this study, MyCB (in Dutch “Koester je lijf”) was adapted and translated for use by Dutch HNC survivors. A forward–backward translation procedure was followed, and texts were revised by a researcher specialized in writing interventions after cancer. Next, MyCB was tested for usability among four HNC survivors. After incorporating their feedback, the adaptation process was completed. MyCB was made available as a booklet and via a website. MyCB is a writing intervention designed to enhance self-compassion toward one’s post-cancer bodily changes, thereby reducing body image-related distress arising from HNC treatment. It entails a self-paced writing activity that is estimated to take 30 min to complete. Participants are initially asked to write freely introducing a negative event related to their changed body after HNC treatment, exploring their deepest thoughts and emotions. Participants then continue writing, guided by written prompts designed to enhance self-compassion toward themselves and their post-cancer body. The prompts encourage participants to practice self-kindness, common humanity, and mindful awareness, consistent with the definition of self-compassion [14].

### Outcome measures

#### Reach of MyCB

The reach of MyCB was calculated by dividing the number of HNC survivors who participated in the intervention study on MyCB, by the total number of (1) eligible HNC survivors for the baseline survey; and (2) HNC survivors who filled in the baseline survey (including those who did not participate in the intervention study).

#### Effects of MyCB

To be able to compare results, the instruments used in the study from Sherman et al. [1] on MyCB among breast cancer survivors were taken over. The primary outcome was body image-related distress. The 10-item Body Image Scale (BIS) [24] measures affective, behavioral, and cognitive body image symptoms and was developed for use in cancer populations. Items can be answered on a 4-point Likert scale ranging from 0 “not at all” to 3 “very much”. A total score (range 0–30) is calculated by summing up the items: a higher score indicates a higher level of body image-related distress. The BIS has shown adequate psychometric properties [25] and is translated and validated in Dutch [26]. Chronbach’s alpha of the BIS in the current study was 0.92.

Secondary outcomes included body appreciation, self-compassion, psychological distress, HRQOL, HNC symptoms, and sexuality. Body appreciation was measured with the 10-item Body Appreciation Scale (BAS-2) [27]. Self-compassion was assessed with the 12-item Self-Compassion Scale–Short Form (SCS-SF) [28]. Psychological distress was measured using the 14-item Hospital Anxiety and Depression Scale (HADS) (total score), and contains two subscales, symptoms of anxiety (HADS-A), and symptoms of depression (HADS-D) [29]. HRQOL was assessed with the summary score of the EORTC QLQ-C30, a cancer-specific quality of life questionnaire [30]. The EORTC QLQ-HN43 is a module specifically designed for HNC patients [31] and was used to measure HNC symptoms. Sexuality was assessed with the 6-item Female Sexual Function Index (FSFI-6) [32] for women and with the 5-item International Index of Erectile Function (IIEF-5) [33] for men. Participants were categorized in the “no sexual activity” group if they reported not to have had sexual activity and intercourse in the past 4 weeks. Validated cut-off scores [32, 33] for women and men were used to characterize participants either as having reported sexual problems or not, to enable cross-gender analyses. Sexuality was not measured at T1, because the FSFI-6 and IIEF-5 assess symptoms from last 4 weeks. All other instruments were measured at T0, T1, and T2. All abovementioned instruments are validated and translated in Dutch. In all instrument validation studies, actions were undertaken to improve readability. For example, adjusting formal words (IIEF-5 and BAS-2) [34, 35], adapting items that were difficult to translate (SCS-SF) [36], or using a translation procedure which makes the items comprehensible to people of all levels of education (EORTC QLQ-C30 and HN43) [30, 31].

#### Factors associated with the reach and with reduced body image-related distress

We investigated factors associated with the reach and with reduced body image-related distress in terms of sociodemographic (age, gender, relationship status, education level, work situation) and clinical characteristics (tumor site, tumor stage, HPV status, time since treatment, treatment modality, surgical reconstruction, neck surgery, extent of surgery). Items on sociodemographic characteristics were included in the T0 survey. Clinical characteristics were retrieved from medical files. Furthermore, T0 scores for body image-related distress, body appreciation, self-compassion, psychological distress, HRQOL, HNC symptoms, and sexuality were analyzed as potential factors associated with the reach.

#### Evaluation of MyCB

In total, 11 study-specific questions in T1 assessed how HNC survivors evaluated MyCB, including reasons to participate (multiple answer options), time investment in MyCB (options ranging from <15 minutes to >2 h), experiences and perceived effects of MyCB, and overall satisfaction with MyCB (scale 0–10). Additionally, HNC survivors answered four open questions about experiences with and recommendations pertaining to MyCB.

### Statistical analyses

The reach and MyCB evaluation questions were explored using descriptive statistics. To investigate which factors are associated with the reach, MyCB participants were compared with non-participants in terms of sociodemographic and clinical factors, body image-related distress (BIS), body appreciation (BAS-2), self-compassion (SCS-SF), psychological distress (HADS total score and subscales), HRQOL (EORTC QLQ-C30 summary score), HNC symptoms (EORTC QLQ-HN43), and sexuality (no sexual activity, sexually active without sexual problems, sexually active with sexual problems). Supplementary file 1 presents the variables and categories. Univariate logistic regression and multiple logistic regression with a stepwise forward selection procedure were applied. Variables were added one by one to the multiple regression model, with *p* value for entry <0.05.

Linear mixed models were used to test the effect of MyCB on body image (BIS total score) and secondary outcomes, except for sexuality (because sexuality is only measured twice). Models included a fixed effect of time and a random intercept for participants. Data were analyzed according to the intention-to-treat principle and all participants (including those who did not make use of MyCB) were approached for T1 and T2. We performed a sensitivity analysis among participants who made use of MyCB. This usage was defined as having at least answered the first prompt (a negative event related to a changed body) and one self-compassion prompt. Secondary outcomes were body appreciation, HRQOL, HNC symptoms that were significantly associated with body image-related distress in a previous study: “problems with social contact” and “problems with wound healing” [8], sexuality, self-compassion, and psychological distress. To assess changes between T0 and T2 in sexual activity and reported sexual problems, McNemar tests were performed.

To identify possible differences in the course of body image-related distress over time between HNC survivors with a BIS score ≥8 and those with a BIS score <8 at baseline, linear mixed models were used, with fixed effects for time, the dichotomized BIS score and their two-way interaction, and a random effect for subject. A significant two-way interaction (*p*<.05) indicates that the change in outcome over time differs between the two groups. A cut-off score of 8 was used, consistent with prior research [37] and to have an acceptable amount of participants in both groups (BIS score of ≥8 and <8).

To investigate factors associated with reduced body image-related distress, univariate logistic regression analysis was applied. HNC survivors who had a clinically relevant reduction of at least 3 points on the BIS between baseline and 1 month post-intervention (i.e., 10% of the instrument range [38]), were compared to those without a 3-point reduction, in terms of sociodemographic and clinical factors.

All analyses used the standard alpha level of 0.05 and were carried out using SPSS version 26 (IBM Corp., Armonk, NY).

### Sample size calculation

In order to show a reduction of 3 points (in accordance with a previous RCT [1]) on the total BIS between T0 and T2, in total, 84 HNC survivors were needed for the intervention study (based on a power of 80% and a significance level of 5%). In this calculation, we anticipated a 20% dropout rate, based on our prior experience conducting intervention studies among HNC survivors [39, 40].

## Results

### Study sample

**Fig. 1:**
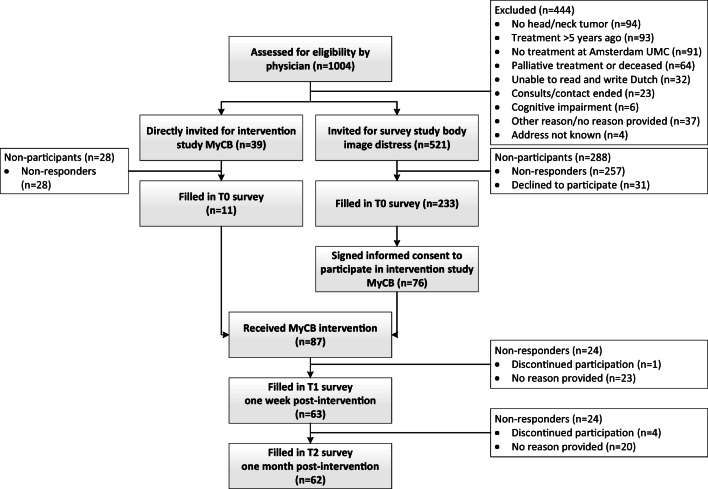
Flow diagram

In total, 521 HNC survivors were invited for a survey on the prevalence of body image-related distress [8], of whom 233 participated (45% response rate) (Fig. 1). Of these 233 HNC survivors, 76 agreed to participate in the intervention study. To achieve the necessary 84 participants, another 39 HNC survivors were directly invited for the MyCB intervention study (and excluded from the reach analysis) of which 11 participated, resulting in a total of 87 HNC survivors. Of those 87 participants, all completed T0, 63 (72%) completed T1, and 62 (71%) completed T2. Patient characteristics are shown in Table 1.

**Table 1:** Patient characteristics.

Characteristic	*n* (%)		
	Participants MyCB (*n*=87)	Participants MyCB (reach analyses) ^a^ (*n*=76)	Non-participants (*n*=157)
Mean age in years [SD]	66 [11.2]	65 [11.8]	68 [10.1]
Gender			
Male	58 (67%)	51 (67%)	103 (66%)
Female	29 (33%)	25 (33%)	54 (34%)
Married/in a relationship^b^			
Yes	63 (72%)	55 (72%)	117 (75%)
No	23 (27%)	21 (28%)	40 (26%)
Education level			
Lower	28 (32%)	26 (34%)	21 (13%)
Middle	39 (45%)	33 (43%)	78 (50%)
Higher	20 (23%)	17 (22%)	58 (37%)
Work situation			
Employed	21 (24%)	19 (25%)	49 (31%)
Unemployed/retired	66 (76%)	57 (75%)	108 (69%)
Tumor site			
Oral cavity	17 (20%)	17 (22%)	34 (22%)
Oropharynx	20 (23%)	17 (22%)	40 (26%)
Hypopharynx	5 (6%)	2 (3%)	10 (6%)
Larynx	29 (33%)	25 (33%)	39 (25%)
Other	16 (18%)	15 (20%)	34 (22%)
Tumor stage ^c^			
I/II	33 (38%)	30 (40%)	73 (47%)
III/IV	47 (54%)	39 (51%)	81 (53%)
HPV positive (oropharyngeal cancer)	14 (70%)	12 (71%)	28 (70%)
Time since treatment, years (median) [IQR]	3.3 [2.5–4.4]	3.3 [2.5–4.6]	3.3 [2.1–4.4]
Single treatment	35 (40%)	31 (41%)	80 (51%)
Surgery	16 (46%)	15 (48%)	47 (49%)
Among which C0–2 laser	11 (69%)	11 (73%)	22 (47%)
Radiotherapy	19 (54%)	16 (52%)	33 (41%)
Combination treatment	52 (60%)	45 (59%)	77 (49%)
Chemoradiotherapy	19 (37%)	16 (36%)	35 (45%)
Surgery and (chemo) radiotherapy	33 (63%)	29 (64%)	42 (55%)
Reconstruction			
None	15 (31%)	15 (34%)	30 (34%)
Primary closure	22 (45%)	18 (41%)	29 (33%)
Surgery with reconstruction	12 (25%)	11 (25%)	30 (34%)
Neck surgery			
Yes	26 (53%)	21 (48%)	41 (46%)
No	23 (47%)	23 (52%)	48 (54%)
Surgery extent			
Small	13 (27%)	13 (30%)	24 (27%)
Moderate	9 (18%)	9 (21%)	21 (24%)
Large	13 (27%)	12 (27%)	24 (27%)
Very large	14 (29%)	10 (23%)	20 (23%)

^a^*n*=11 patients were excluded for the reach analysis, because they were directly invited for the MyCB intervention study. ^b^ *n*=1 missing in participants MyCB. ^c^ *n*=7 missing in participants MyCB and *n*=3 missing in non-participants

### Reach of MyCB

The reach was 15% (76 participants out of 521 eligible HNC survivors) to 33% (76 participants out of 233 responders). In total, 59% of the participants chose the booklet and 41% chose the website. Factors associated with the reach of MyCB in the univariate and multivariate analysis are shown in Supplementary file 1. Factors that were significantly associated with the reach of MyCB in the multivariate analysis, were education level (*p*=0.001), social eating problems (*p*=0.003) and wound healing problems (*p*=0.041). MyCB was more likely to reach HNC survivors who were lower educated (reference category) than middle or higher educated HNC survivors. MyCB was also more likely to reach HNC survivors with more social eating problems and HNC survivors with fewer wound healing problems. The model explained 15% (Nagelkerke R^2^) of the variance in reach.

### Effects of MyCB

**Fig. 2:**
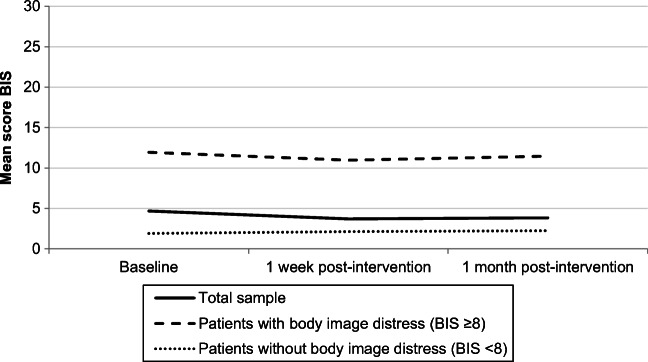
The course of body image-related distress of the total sample (*n*=87); participants with BIS score ≥8 (*n*=24) and participants with BIS score <8 (*n*=63)

Nine HNC survivors (10%) showed a clinically relevant improvement in body image-related distress of 3 points between baseline and 1 month post-intervention. Across all 87 participants, the difference in BIS mean scores compared to the baseline score was not statistically significant at 1 week (*p*=0.89) and 1 month (*p*=0.73) post-intervention. The sensitivity analysis among MyCB users (*n*=41) showed also no significant effect on body image-related distress. The course of body image-related distress over time was not significantly different (*p*=0.38) between HNC survivors with a BIS score ≥8 (*n*=24) and those with a BIS score <8 (*n*=63) (Fig. 2). Self-compassion improved significantly during follow-up until 1 month post-intervention (*p*=0.003). No significant effects were observed on other secondary outcomes (Table 2). No factors were associated with reduced body image-related distress (Supplementary file 2).

**Table 2:** Descriptives and linear mixed model analyses at baseline (T0), 1 week- (T1), and 1 month (T2) post-intervention

	Descriptives	Linear mixed model analysis		
	Mean (SD)	Estimated mean change from baseline	95% CI	Sig.
Body image-related distress (range 0–30)				0.89
T0	4.7 (5.4)	n/a^a^		
T1	3.7 (4.8)	−0.1	−0.8 to 0.7	
T2	3.9 (4.8)	0.1	−0.6 to 0.9	
Sensitivity analysis MyCB users				0.62
T0	3.1 (3.8)	n/a		
T1	2.9 (3.2)	−0.2	−1.1 to 0.6	
T2	3.1 (3.8)	0.1	−0.7 to 1.0	
Body appreciation (range 1–5)				0.43
T0	4.0 (0.7)	n/a		
T1	4.1 (0.6)	0.1	0.0 to 0.1	
T2	4.0 (0.6)	0.0	−0.1 to 0.1	
Self-compassion (range 1–7)				**0.009**
T0	4.7 (0.8)	n/a		
T1	5.0 (1.0)	0.2	0.0 to 0.3	
T2	5.1 (1.0)	0.2	0.1 to 0.4	
Psychological distress (range 0–42)				0.67
T0	10.8 (7.9)	n/a		
T1	9.2 (7.2)	−0.3	−1.1 to 0.5	
T2	10.0 (7.5)	0.1	−0.8 to 0.9	
Health-related quality of life (range 0–100)				0.84
T0	79.8 (16.6)	n/a		
T1	82.0 (13.6)	−0.1	−0.3 to 0.2	
T2	81.4 (15.2)	−0.1	−0.3 to 0.2	
Problems with social contact (range 0–100)				0.07
T0	8.4 (22.3)	n/a		
T1	9.5 (21.1)	0.3	−0.1 to 0.7	
T2	4.8 (13.3)	−0.2	−0.6 to 0.2	
Problems with wound healing (range 0–100)				0.78
T0	7.4 (18.0)	n/a		
T1	8.1 (19.7)	0.1	−0.4 to 0.6	
T2	6.4 (15.8)	0.0	−0.5 to 0.5	
Sexually active (yes/no)				McNemar Test (*n*=54) 0.77
T0 (*n*=79)	Yes *n*=43 (54%) No *n*=36 (46%)			
T2 (*n*=57)	Yes *n*=23 (40%) No *n*=34 (60%)			
Reported sexual problems among sexually active patients (yes/no)				McNemar Test (*n*=18) 1.00
T0 (n=43)	Yes *n*=24 (56%) No *n*=19 (44%)			
T2 (n=23)	Yes *n*=13 (57%) No *n*=10 (43%)			

Significant differences (p<0.05) are presented in bold font. ^**a**^ not applicable

### Evaluation MyCB

Table 3 presents the MyCB evaluation results. In summary, HNC survivors primarily participated because they were asked to (for the sake of research) (89%). Almost half of the participants spent between 15 and 30 min undertaking the writing activity (49%). In the writing activity, the majority (78%) was able to express concerns regarding their body or appearance “quite a bit” or “very much”. Most participants were positive about the writing activity and found it clear, complete, meeting expectations, useful, and clarifying. A small group reported that the writing activity was “quite a bit” or “very much” confronting (31%), or bothersome (12%). The most reported value of the writing activity was that they learned that other people also have body distress (33%). In total, 42% of participants reported having gained insights to deal with body/appearance after cancer. In the open-ended questions, participants shared their thoughts on the added value of the writing activity, gained insights, unnecessary/missed parts, and additional tips. The writing activity was rated with a 7.2 on a scale of 0–10 for satisfaction.

**Table 3:** Answers to the evaluation questions of MyCB

Questions and answer options	*n*	%	Open answers
1. What was the (most important) reason to participate in this research? (multiple answers possible)			
I was asked to participate in this research	56	89%	
I wanted to tell my story	11	18%	
To feel better about my body/appearance	3	5%	
Other reasons	12	19%	
2. How much time did you spend to the writing activity?			
Less than 15 min	9	10%	
Between 15 and 30 min	30	49%	
Between 30 min and 1 h	18	30%	
Between 1 h and 1.5 h	6	10%	
Between 1.5 h and 2 h	0	0%	
More than 2 h	1	2%	
3. In the writing activity, were you able to express everything that you were concerned about regarding your body/appearance?			
Not at all	1	2%	
A little	12	20%	
Quite a bit	26	44%	
Very much	20	34%	
4a. Did you find the writing activity clear?			
Not at all	4	7%	
A little	10	17%	
Quite a bit	34	58%	
Very much	11	19%	
4b. Did you find the writing activity complete?			
Not at all	3	5%	
A little	10	18%	
Quite a bit	30	54%	
Very much	13	23%	
4c. Did the writing activity meet your expectations?			
Not at all	4	7%	
A little	11	19%	
Quite a bit	32	55%	
Very much	9	16%	
4d. Did you find the writing activity useful?			
Not at all	3	5%	
A little	13	22%	
Quite a bit	26	45%	
Very much	16	28%	
4e. Did you find the writing activity clarifying?			
Not at all	8	14%	
A little	11	19%	
Quite a bit	26	45%	
Very much	13	22%	
4f. Did you find the writing activity confronting?			
Not at all	25	42%	
A little	16	27%	
Quite a bit	11	18%	
Very much	8	13%	
4g. Did you find the writing activity bothersome?			
Not at all	40	68%	
A little	12	20%	
Quite a bit	4	7%	
Very much	3	5%	
5. What do you think is the added value of the writing activity? (multiple answers possible)			
I better understand feelings about my body and my appearance	6	10%	
I am better able to distance myself from my feelings, thoughts and/or behavior about my body	10	17%	
I have become kinder to myself and my body	7	12%	
I know that other people have similar experiences (for example, not feeling comfortable about their appearance or body)	20	33%	
None of the above	19	32%	
Other comments	14	22%	• “I realized that I can trust my body if something is ‘wrong’, my body gives me a clear signal.” • “No matter how much you write compassionately about your body/defects, they will not come back.” • “Advantage: writing about what concerns you unconsciously. Disadvantage: being confronted with what has happened, reliving it. Trying to clear your head, also from things that have nothing to do with cancer.” “The writing activity is about people’s opinion. Personally I prefer facts.”
6. As a result of the writing activity, did you gain insight(s) for dealing with your body / appearance after cancer?			
Yes	23	42%	
No	32	58%	
7. Can you describe which insight(s) you have received?			• “Be kind to yourself. Accept your body as it is. You’re still the same person. Appearance is inferior. Be yourself.” • “That I have constant pain and fatigue and that I’ve become insecure.” • “That [after the treatment] I am a healthy and privileged person.”
8. Did you find certain parts unnecessary and, if so, which?			• “It was not applicable to my situation.” • “I have no changed appearance, so the questions were difficult to answer.” • “I found the prompts too vague. Shorter, more guided questions would be more effective. It was multi-interpretable now.”
9. Have you missed any parts and, if so, which ones?			• “The questions are too general. I had a tumor in my throat and therefore problems with swallowing and taste.” • “Questions about a changed diet.” • “Questions about a voice prosthesis.” • “Behavior change. I would like to learn how to get angry and how to take care of myself.” • “How I experience my rehabilitation process, is it taking too long?” • “A clear description of the patients’ perspective with regard to his past.”
10. Do you have any additional tips and/or comments?			• “It was a pleasant activity for me, to fill in the writing activity. It gives you a moment of reflection on all events. The entire cancer trajectory passes you by like a rollercoaster. A moment of reflection.” • “It seems to me that the writing activity in this form is not suitable for laryngectomized patients. This is due to the relatively difficult formulation of the questions asked.” • “It has not changed anything for my acceptance / well-being. I struggle daily with the consequences! I am trying to enjoy life but it is not easy.” • “I would opt for a more guiding way of asking. This was far too open-ended and therefore not stimulating enough to achieve true self-reflection.”
11. In sum, how do you grade the writing activity? 0: very poor to 10: very good (mean, SD)	7.2 (1.5)		

## Discussion

This pretest–posttest study investigated the reach of the structured writing activity MyCB among HNC survivors and its effect on body image-related distress. The reach of MyCB was 15–33%. MyCB especially reached HNC survivors with a lower education, more social eating problems, and fewer wound healing problems. No significant change in body image-related distress between baseline and post-intervention was found, nor in body appreciation, psychological distress, HRQOL, HNC symptoms, and sexuality. Self-compassion significantly increased between baseline and 1 month post-intervention.

The reach of MyCB (15–33%) fell within the expected range (13–24%) [10, 22, 23], and the upper range is higher. A possible explanation for the higher upper range is that abovementioned studies have explored the need for care regarding body image, which provides only an indication for the actual reach of a body image intervention. Also, HNC survivors have a preference for written material as a source of supportive care for body image-related distress (like MyCB), when compared to counseling, support groups, information via the computer, or referral to a mental health specialist [10].

As expected, higher body image-related distress was univariately associated with the reach of MyCB. However, other factors were more strongly associated with the reach in the multivariable analysis. MyCB especially reached lower educated HNC survivors, which is a positive finding given the fact that studies on psychosocial interventions tend to mostly reach highly educated cancer patients [41]. This might be related to the fact that participants could choose a booklet version instead of a website, since lower educated cancer patients are less likely to use the internet [42].

The absence of change in body image-related distress did not support our hypothesis that MyCB would reduce body image-related distress in HNC survivors, nor the findings from a previous RCT on MyCB among breast cancer survivors [1]. This might be explained by the low level of body image-related distress pre-intervention: a mean BIS score of 4.7. This is in contrast with the RCT (mean BIS score 11.5), where women were only included if they experienced at least one negative event related to bodily changes after breast cancer. The absence of change may be caused by a floor effect [43]. However, we compared HNC survivors with a BIS score ≥8 to those with a BIS score <8 and found no significant difference in the course of body image-related distress, which indicates that a floor effect is no plausible explanation.

Another explanation for the absence of change may be the difference in body image symptoms between breast cancer and HNC survivors. For HNC survivors, damaged essential body functions like speech and swallowing with a large impact on social life are central aspects of body image-related distress [8]. Breast cancer and its treatment does not impair essential body functions as profoundly, so disfigurement may be a more central aspect of body image. Possibly, self-compassion positively influences thoughts and feelings related to disfigurement (attractiveness, appearance: “Looks aren’t everything”; “I’m more than my body”) but not thoughts and feelings related to dysfunction in speech and swallowing.

Results showed that MyCB has a positive influence on self-compassion. This is consistent with the previous RCT among breast cancer survivors [1]. In that RCT, the significant effect of MyCB on body image-related distress was mediated by self-compassion. It was suggested that a high level of self-compassion would be a protective factor for breast cancer survivors at risk of experiencing body image-related distress. However, this technique does not seem to apply to HNC survivors (because we found no effect on body image-related distress).

HNC survivors rated their satisfaction with MyCB as 7.2/10. Additional results showed that HNC survivors were generally positive about the writing activity, with 78% indicating they were able to express everything they were concerned about regarding their body/appearance. By contrast, 58% indicated they did not gain insights in dealing with body/appearance changes after cancer, possibly related to difficulties that some HNC survivors indicated in interpreting the guided self-compassion prompts within the context of their specific treatment. For HNC survivors, the writing activity would likely need to be modified to better reflect the functional bodily changes following HNC treatment, rather than appearance changes. The impact of physical dysfunction on social contact suggests that people might benefit from a combination of the writing activity with practical strategies to cope with e.g., rejection, stigma, shame or frustration. Moreover, the most reported value of the writing activity was learning that other people also have body image issues (33%), which suggests that people might benefit from a group intervention format.

A limitation of this study is that we built on the previous RCT [1] among breast cancer survivors, and did not include a control group to compare outcomes in our study. Another limitation is that relatively few people with high body image-related distress (i.e., BIS scores of ≥8) participated, which might have attenuated any effect of the writing activity. However, a sub-analysis among high scoring HNC survivors showed no effect. Lastly, this was a single-center study, in one country. Therefore, results should be interpreted with caution, and we can only conclude that it is likely that MyCB is effective in HNC survivors to improve self-compassion.

For the purpose of alleviating body image-related distress in HNC survivors, MyCB in its current form is not the preferred intervention of choice due to the absence of an effect. However, the writing activity can be useful to improve self-compassion in HNC survivors. Having a kind and non-judgmental perspective toward oneself and recognizing that suffering is part of the shared human experience, may provide some alleviation to the burden of cancer [44].

Due to the paucity of evidence-based interventions to reduce body image-related distress in HNC survivors, more research is needed to develop and investigate body image interventions. It is hypothesized that HNC survivors might benefit from interventions that focus on coping with speech and swallowing problems, especially in social situations, as body image-related distress in HNC survivors is mainly caused by (social) difficulties resulting from these physical dysfunctions [8].

## Conclusion

In conclusion, MyCB reached up to a third of HNC survivors, especially those with a lower education, more social eating problems, and fewer wound healing problems. MyCB did not reduce body image-related distress, but is likely to improve self-compassion sustaining up to 1 month after intervention use.

## Supplementary Information

ESM 1 (DOCX 16 kb)

ESM 2 (DOCX 13 kb)

## Data Availability

N/A.

## Code availability

N/A.
