# Supplementary material for: A structured expressive writing activity targeting body image-related distress among head and neck cancer survivors: who do we reach and what are the effects?
Source: Support Care Cancer. 2021 Mar 18;29(10):5763–76. doi: 10.1007/s00520-021-06114-y (PMC8410700; doi:10.1007/s00520-021-06114-y)
Supplement: Supplementary file 1 — (DOCX 16 kb) [file 520_2021_6114_MOESM1_ESM.docx]

**Supplementary file 1. Univariate and multivariate regression of factors associated with the reach, and descriptive statistics.**

Manuscript title: A structured expressive writing activity targeting body image-related distress among head and neck cancer patients: who do we reach and what are the effects?

Journal: Supportive Care in Cancer

Authors: H.C. Melissant, F. Jansen*, S.E.J. Eerenstein, P. Cuijpers, B.I. Lissenberg-Witte, K.A. Sherman, E. Laan, C.R. Leemans, I.M. Verdonck-de Leeuw

*Corresponding author:

Femke Jansen

Amsterdam UMC, Vrije Universiteit Amsterdam, Department of Otolaryngology-Head and Neck Surgery, P.O. Box 7057, 1007 MB Amsterdam, Netherlands
Tel: +31 20 444 0681

E-mail: f.jansen1@amsterdamumc.nl

|  | Univariate | | Multivariate | |
| --- | --- | --- | --- | --- |
| Variable | OR [95% CI] | Sig. | OR [95% CI] | Sig. |
| Age | 0.98 [0.95-1.00] | 0.10 |  |  |
| Gender |  | 0.82 |  |  |
| Female | 1 |  |  |  |
| Male | 1.1 [0.60-1.9] |  |  |  |
| Married/ in a relationship |  | 0.73 |  |  |
| Yes | 1 |  |  |  |
| No | 1.1 [0.60-2.1] |  |  |  |
| Education level |  | **0.001** |  | **0.001** |
| Lower | 1 |  | 1 |  |
| Middle | 0.34 [0.17-0.69] |  | 0.32 [0.15-0.69] |  |
| Higher | 0.23 [0.11-0.52] |  | 0.23 [0.11-0.52] |  |
| Work situation |  | 0.38 |  |  |
| Employed | 1 |  |  |  |
| Unemployed/retired | 1.3 [0.71-2.5] |  |  |  |
| Tumor site |  | 0.59 |  |  |
| Oral cavity | 1 |  |  |  |
| Oropharynx | 0.85 [0.38-1.9] |  |  |  |
| Hypopharynx | 0.40 [0.08-2.0] |  |  |  |
| Larynx | 1.3 [0.59-2.8] |  |  |  |
| Other | 0.88 [0.38-2.1] |  |  |  |
| Tumor stage |  | 0.59 |  |  |
| I/II | 1 |  |  |  |
| III/IV | 1.2 [0.66-2.1] |  |  |  |
| Time since treatment | 1.1 [0.89-1.3] | 0.44 |  |  |
| Treatment modality |  | 0.24 |  |  |
| Surgery | 1 |  |  |  |
| Radiotherapy | 1.5 [0.66-3.5] |  |  |  |
| Chemoradiotherapy | 1.4 [0.63-3.3] |  |  |  |
| Surgery plus (chemo)radiotherapy | 2.2 [1.02-4.6] |  |  |  |
| Surgery extent ^a^ |  | 0.98 |  |  |
| Very large | 1 |  |  |  |
| Large | 1.00 [0.36-2.8] |  |  |  |
| Moderate | 0.86 [0.29-2.6] |  |  |  |
| Small | 1.1 [0.39-3.0] |  |  |  |
| Reconstruction |  | 0.52 |  |  |
| None | 1 |  |  |  |
| Primary closure | 1.2 [0.53-2.9] |  |  |  |
| Surgery with reconstruction | 0.73 [0.29-1.7] |  |  |  |
| Neck surgery |  | 0.86 |  |  |
| No | 1 |  |  |  |
| Yes | 1.1 [0.52-2.2] |  |  |  |
| HPV ^b^ |  | 0.69 |  |  |
| Negative | 1 |  |  |  |
| Positive | 0.77 [0.21-2.8] |  |  |  |
| Body image distress | 1.1 [1.01-1.1] | **0.018** |  |  |
| Body appreciation | 0.99 [0.95-1.03] | 0.49 |  |  |
| Quality of life^c^ | 0.73 [0.60-0.89] | **0.002** |  |  |
| HNC symptoms^c^ |  |  |  |  |
| Fear of progression | 1.2 [1.04-1.3] | **0.011** |  |  |
| Dry mouth and sticky saliva | 1.1 [0.97-1.2] | 0.20 |  |  |
| Pain in the mouth | 1.2 [1.04-1.4] | **0.012** |  |  |
| Problems with senses | 1.05 [0.95-1.2] | 0.38 |  |  |
| Problems with shoulder | 1.00 [0.88-1.1] | 0.94 |  |  |
| Skin problems | 1.1 [0.95-1.3] | 0.20 |  |  |
| Social eating | 1.2 [1.1-1.3] | **0.003** | 1.2 [1.08-1.4] | **0.003** |
| Speech | 1.1 [1.02-1.2] | **0.024** |  |  |
| Swallowing | 1.2 [1.04-1.3] | **0.010** |  |  |
| Problems with teeth | 1.2 [1.02-1.3] | **0.021** |  |  |
| Coughing | 1.1 [1.01-1.2] | **0.040** |  |  |
| Swelling in the neck | 1.2 [1.02-1.3] | **0.023** |  |  |
| Neurological problems | 1.1 [0.97-1.2] | 0.20 |  |  |
| Trismus | 1.1 [0.99-1.2] | 0.07 |  |  |
| Social contact | 1.1 [0.96-1.4] | 0.15 |  |  |
| Weight loss | 1.04 [0.93-1.2] | 0.52 |  |  |
| Problems with wound healing | 0.99 [0.85-1.1] | 0.84 | 0.83 [0.69-0.99] | **0.041** |
| Psychological distress | 1.04 [1.00-1.1] | 0.052 |  |  |
| Symptoms of depression | 1.1 [0.98-1.1] | 0.13 |  |  |
| Symptoms of anxiety | 1.1 [1.00-1.1] | **0.040** |  |  |
| Self-compassion | 0.86 [0.64-1.2] | 0.32 |  |  |
| Sexuality |  | 0.97 |  |  |
| No sexual activity | 1 |  |  |  |
| Sexually active without sexual problems | 0.95 [0.46-1.9] |  |  |  |
| Sexually active with sexual problems | 0.92 [0.47-1.8] |  |  |  |

EORTC QLQ-C30/H*N43* 30-item core European Organisation for Research and Treatment of Cancer Quality of Life Questionnaire/head and neck cancer, 43 items, *HADS* Hospital Anxiety and Depression Scale, *SCS-SF* Self Compassion Scale – Short Form. ^a^ Small: C02-laser of vocal fold, lip excision, ear amputation, skin excision small nose tumor. Moderate: excision of sublingual/submandibular salivary gland, transoral excision, lip surgery with reconstruction, partial sinus resection, skin excision with local reconstruction, neck surgery. Large: parotidectomy with neck surgery, marginal and segmental mandibular resection, transoral excision with reconstruction, extensive sinus surgery, maxillectomy, skin excision with neck surgery or reconstruction. Very large: commando procedure, laryngectomy, lateral temporal bone surgery. ^b^ n=54 oropharyngeal cancer patients with a known HPV status. ^c^ OR per 10 point increase in subscale. Significant differences (p<0.05) are presented in bold font.
