# Supplementary material for: A structured expressive writing activity targeting body image-related distress among head and neck cancer survivors: who do we reach and what are the effects?
Source: Support Care Cancer. 2021 Mar 18;29(10):5763–76. doi: 10.1007/s00520-021-06114-y (PMC8410700; doi:10.1007/s00520-021-06114-y)
Supplement: Supplementary file 2 — (DOCX 13 kb) [file 520_2021_6114_MOESM2_ESM.docx]

**Supplementary file 2. Univariate regression analysis of factors associated with reduced body image-related distress (improvement of 3 points or more).**

Manuscript title: A structured expressive writing activity targeting body image-related distress among head and neck cancer patients: who do we reach and what are the effects?

Journal: Supportive Care in Cancer

Authors: H.C. Melissant, F. Jansen*, S.E.J. Eerenstein, P. Cuijpers, B.I. Lissenberg-Witte, K.A. Sherman, E. Laan, C.R. Leemans, I.M. Verdonck-de Leeuw

*Corresponding author:

Femke Jansen

Amsterdam UMC, Vrije Universiteit Amsterdam, Department of Otolaryngology-Head and Neck Surgery, P.O. Box 7057, 1007 MB Amsterdam, Netherlands
Tel: +31 20 444 0681

E-mail: f.jansen1@amsterdamumc.nl

| Variable | OR [95% CI] | Sig. |
| --- | --- | --- |
| Age |  | 0.13 |
| <67 | 1 |  |
| ≥67 | 0.29 [0.06-1.5] |  |
| Gender |  | 1.00 |
| Female | 1 |  |
| Male | 1.00 [0.23-4.3] |  |
| Married/ in a relationship |  | 0.29 |
| Yes | 1 |  |
| No | 0.31 [0.04-2.7] |  |
| Education level |  | 0.94 |
| Lower | 1 |  |
| Middle/higher | 0.94 [0.22-4.1] |  |
| Work situation |  | 0.37 |
| Employed | 1 |  |
| Unemployed/retired | 0.5 [0.11-2.3] |  |
| Tumor site ^a^ |  | 0.14 |
| Oral cavity/oropharynx | 1 |  |
| Hypopharynx/larynx | 0.19 [0.02-1.8] |  |
| Tumor stage |  | 0.34 |
| I/II | 1 |  |
| III/IV | 2.3 [0.43-12.0] |  |
| Time since treatment |  | 0.26 |
| <3 years | 1 |  |
| ≥3 years | 2.6 [0.50-13.2] |  |
| Treatment modality |  | 0.09 |
| Single treatment | 1 |  |
| Combination treatment | 6.2 [0.74-51.8] |  |

^a^ Other tumor sites are excluded from the analysis.
